# Digital media pattern design compression and optimization method based on K-means clustering and LLE dimensionality reduction

**DOI:** 10.1371/journal.pone.0350623

**Published:** 2026-06-16

**Authors:** Binmei Liu, Shiwan Zhou, Jing Sun, Xiaojuan Liu, Wenting Lu

**Affiliations:** 1 The Academy of VR and Art, Jiangxi University of Software Professional Technology, Nanchang, China; 2 College of Creative Arts, Universiti Teknologi MARA, Selangor, Malaysia; 3 College of Humanities, Gandong University, Fuzhou, China; 4 Postdoctoral Research Station Pioneer Software Inc, Jiangxi University of Software Professional Technology, Nanchang, China; PLOS, UNITED KINGDOM OF GREAT BRITAIN AND NORTHERN IRELAND

## Abstract

The rapid development of digital media demands higher quality in pattern design. Simultaneously, significant redundant information hinders the transmission and sharing of these patterns, creating an urgent need for image compression. However, traditional image compression methods struggle to balance efficiency and quality. To address this, a new image compression model for digital media patterns is proposed, based on K-means clustering and Locally Linear Embedding methods. This model creates an efficient compression solution by integrating dynamic clustering parameter selection based on color histograms, multi-dimensional image segmentation of color and texture, and a local linear embedding dimensionality reduction algorithm using dynamic neighborhood selection. The model achieves a compression ratio of 84% and a Peak Signal-to-Noise Ratio of 41dB, with no significant quality difference before and after compression, validating the effectiveness of the improvements made to the basic methods. In practical application experiments, the model’s multi-scale structural similarity reaches 0.71. When processing a large number of patterns, the fastest response time is 189ms, with a minimum memory usage of 16.1M. The shortest processing time on a single-core processor is 0.34s. These experimental results demonstrate that the model balances compression efficiency and quality, offering superior compression performance, good robustness, and the ability to handle various complex tasks and adapt to different application scenarios, meeting the high standards of the digital media industry for image compression.

## 1. Introduction

With the development of the internet and the iteration of various graphic design software, digital media patterns have not only improved in quality but also become more content-rich, while facing storage and transmission issues. Digital media patterns are visual elements in digital media art, typically defined as graphics or decorative motifs created via digital technology with specific design intentions, including but not limited to ordered combinations of visual elements such as color composition, texture features, shape layout, etc. Digital media patterns are transformed, reorganized, and integrated while respecting the symbolic meaning of the original image to better align with modern aesthetics and market demand. Their high resolution and complex designs provide users with a strong visual impact [1–2]. However, these characteristics impose higher demands on image storage and transmission, requiring high-quality compression of the original patterns to ensure smooth transmission and sharing of digital media patterns [3]. The large volume of data and the presence of redundant data make the storage and transmission of digital media patterns difficult and inefficient [4]. To improve storage and transmission efficiency, save bandwidth and energy, and enhance user experience, compression and optimization of digital media patterns are necessary. However, traditional image compression methods achieve smaller image sizes at the cost of image quality [5–6]. Huang et al. proposed an image data semantic communication model that utilizes reinforcement learning and adaptive semantic coding to perform pixel-level image encoding and reconstruction. The results indicate that the model has noise resistance and can compress and reconstruct images under low bit rate conditions [7]. To address the hardware resource burden caused by hyperspectral pattern processing and storage, Zhang et al. proposed an unsupervised dimensionality reduction method based on dynamic mode decomposition. The method showed good performance in terms of average peak signal-to-noise ratio and average principal angle mapping in comparative experiments, proving it to be a useful tool [8]. To balance the high compression ratio and image quality in medical image processing, and alleviate pressure on storage capacity and hardware devices, Dimililer et al. trained machine learning algorithms to ensure both compression ratio and image quality. Experimental results showed that the radial basis function neural network can be effectively used for classifying the optimal compression ratio, achieving a highest recognition rate of 90.625% [9]. Duan et al. proposed a new lossy image compression scheme based on variational autoencoders to address the lossy image compression issue. This scheme enables efficient decoding and variable-rate compression, with numerous experimental results indicating significant advantages in compression rate and rate-distortion performance over existing benchmark methods [10].

The K-means clustering algorithm is a widely used clustering analysis method due to its simplicity, speed, and broad applicability. It has become a favored research topic among experts and scholars in various fields. For example, Nie et al. re-expressed the objective function of the traditional K-means clustering algorithm as a maximization problem, reducing the need for additional intermediate variables and lowering computational complexity, thereby alleviating pressure on computer resources [11]. To achieve image compression in environments with limited transmission bandwidth and storage capacity, Liang et al. proposed a compression algorithm that combines the K-means clustering algorithm with neural networks. The results showed that compared with traditional neural network algorithms, the proposed algorithm improved the running time by 9.5% [12]. Zhao et al. proposed a lossless encryption method based on dual images, which introduces the characteristics of memristors into four-dimensional cellular neural networks and obtains partial keys through the K-means to make the keys more random. The results show that the proposed method exhibits good encryption performance in various indicators such as key sensitivity and information entropy [13].

Locally Linear Embedding (LLE), known for its ability to handle high-dimensional data, has been studied and optimized by scholars both domestically and internationally. For example, Lee et al. proposed a method for detecting abnormalities in the manufacturing industry based on the LLE algorithm, addressing the limitations of data utilization caused by imbalanced data. This method clusters the normal data distribution using the LLE algorithm to label different types of anomalies. Comparative experiments confirmed the superiority of this method in dimensionality reduction, with an F-1 score of 0.86 [14]. Chu et al. combined kernel-based LLE and partial least squares to fully exploit both local and global data information in a dataset, proposing a new machine operation performance evaluation method. Experiments showed that this method had significant advantages in detecting machine issues efficiently, helping producers adjust machines in time to maximize overall economic benefits [15].

In summary, experts and scholars in various fields have explored and researched image compression processing and optimization, with applications already realized in some areas. However, existing methods still have shortcomings in balancing compression quality and computational efficiency, especially in traditional K-means clustering, which heavily relies on manually setting parameters and has randomness in initial center selection. Meanwhile, traditional LLE algorithms are sensitive to neighborhood parameters and exhibit instability on high-dimensional feature data. In response to the above issues, this study proposes a K-means LLE compression model that combines dynamic parameter selection with multi-dimensional feature fusion, filling the research gap in parameter adaptive optimization, multi-dimensional feature complementary utilization, and local structure preservation for dimensionality reduction in image compression. The research aims to construct a compression model that can adaptively handle digital media modes, achieve a better balance between compression ratio and visual quality, and promote efficient storage, fast transmission, and intelligent processing of digital media resources.

The innovation of this study lies in: (1) proposing a clustering center initialization method based on geometric principles, and dynamically determining the number of clusters using color histograms to improve the stability and clustering quality of K-means, reducing the dependence on manually set parameters. (2) improving the nearest neighbor selection mechanism of the LLE algorithm, introducing class sample information to enhance the dimensionality reduction effect, and adaptively adjusting the neighborhood size based on local curvature measurement. (3) building an end-to-end compression model that integrates improved K-means and LLE algorithms to achieve high-quality compression and efficient dimensionality reduction.

## 2. Methods and materials

### 2.1. Design of pattern compression method based on k-means clustering

**Fig 1:**
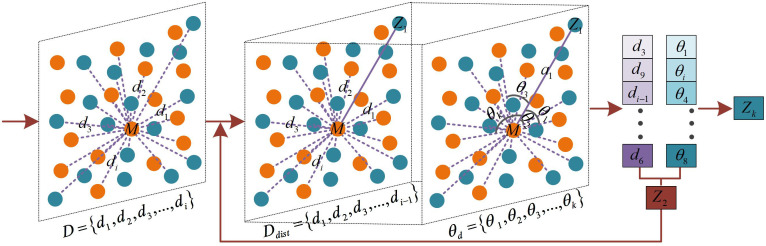
Initialization of cluster centers based on geometric principles.

K-means represents the entire image using fewer colors, thereby achieving rapid image compression. The process of compressing an image using K-means involves first converting the image into a specific one-dimensional array, with each pixel represented as an element. Then, the clustering algorithm is applied to these one-dimensional arrays, clustering them into a fixed number of clusters. Based on the algorithm’s results, the centroid of each cluster is located, and the color of the centroid replaces the original pixel colors in the image. Finally, the compressed pixels are reconstructed to obtain the compressed image. K-means segmentation is simple and independent of dataset size. However, since the initial cluster centers are selected randomly, the quality of the clustering results depends heavily on the selection of these centers, leading to potential instability. Therefore, this study selects initial cluster centers using Euclidean distance and vector angle parameters, prioritizing points that maximize both inter-cluster distance and centroid vector angle differences (Fig 1).

As seen in Fig 1, the algorithm for initializing cluster centers based on geometric principles first reduces the dimensionality of the dataset, transforming high-dimensional data into a two-dimensional dataset. The mean center of the dataset, denoted by M, is then computed, and the distances $D=\{d_{\text{1}},d_{\text{2}},d_{\text{3}},...,d_{i}\}$ from each cluster to the mean center are calculated. The cluster $Z_{\text{1}}$ with the largest distance is chosen as the first initialization center. The vector between the first initialization center and the mean center is considered the reference vector $a_{\text{1}}$, and the Euclidean distance M between the mean center $D_{dist}=\{d_{\text{1}},d_{\text{2}},d_{\text{3}},...,d_{i-1}\}$ and the other clusters, excluding the first initialization center, is calculated. The angles formed by the three points—the mean center M, the first initialization center $Z_{\text{1}}$, and all other clusters—are calculated to form the angle set $\theta_{d}=\{\theta \text{1},\theta_{\text{2}},\theta_{\text{3}},...,\theta_{k}\}$. Both the distance set $D_{dist}$ and the angle set $\theta_{d}$ are sorted in descending order, and the cluster with the smallest combined rank of distance and angle is selected as the next initialization center $Z_{\text{2}}$. This process is repeated until k initialization centers are found. The vector between two clusters is calculated as shown in Equation (1).


$$\begin{array}{c} a=\left(x_{i1},x_{j1}\right)-\left(x_{i2},x_{j2}\right) \end{array}$$ (1)

In Equation (1), $x_{\text{1}}$ and $x_{\text{2}}$ represent two different clusters. The Euclidean distance between the two vectors is obtained using Equation (2).


$$\begin{array}{c} d\left(x_{i},x_{j}\right)=({\left|x_{i1}-x_{j1}\right|}^{\text{2}}+,...,+{\left|x_{ip}-x_{jp}\right|}^{\text{2}}{)}^{\frac{\text{1}}{\text{2}}} \end{array}$$ (2)

In Equation (2), $x_{i}$ and $x_{j}$ represent two p -dimensional clusters. The angle between the two vectors is calculated using Equation (3).


$$\begin{array}{c} \theta \left(a_{\text{1}},a_{\text{2}}\right)=arccos\frac{a_{\text{1}}a_{\text{2}}}{\left|a_{\text{1}}\right|\left|a_{\text{2}}\right|} \end{array}$$ (3)

**Fig 2:**
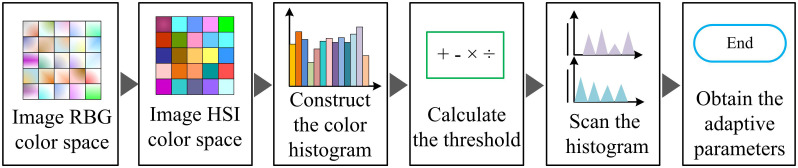
Dynamically determining the number of clusters based on the color histogram.

In Equation (3), $a_{\text{1}}$ and $a_{\text{2}}$ represent two vectors. Using geometric principles to select cluster centers can reduce the impact of random cluster center selection on the clustering results and yield better clustering performance. However, the selection of the k value still depends on manual input, requiring the user to have a high level of expertise to select the appropriate k value. Color histograms quantify the colors in an image, enabling the statistical analysis of the image’s color features [16–17]. This technique adapts well to various image variations and provides robust global feature descriptions. It has been successfully applied in fields such as photographic post-processing and computer vision [18]. Therefore, the study introduces color histograms to dynamically determine the number of clusters that best suit the algorithm (Fig 2). Color histograms are used to dynamically determine the number of clusters k by combining the image’s color features.

As shown in Fig 2, the first step in dynamically determining the number of clusters based on color histograms is to convert the image’s RGB colors to the Hue-Saturation-Intensity (HSI) color space. After the color transformation, the colors are quantized, grouping similar colors into a primary color, yielding the color feature values. A color histogram is built based on these values. After determining the threshold for the number of clusters, a vertical and horizontal scan is performed to find the actual peak values of the color features. Among them, vertical scanning refers to traversing each color interval of the histogram and selecting intervals with count values higher than the preset minimum peak height threshold as candidate peaks. Horizontal scanning refers to checking the interval distance between adjacent peaks in the candidate peaks. If it is less than the set minimum distance, the lower peaks are merged or removed, and the final number of retained peaks is the adaptively determined cluster number k. The peak detection process sets a minimum peak height threshold equal to 15% of the histogram’s maximum value. It requires a minimum distance of 5 histogram intervals between peaks to avoid false peaks caused by noise. The number of reliable peaks identified through this process corresponds to the final number of clusters k, achieving adaptive partitioning of image color complexity. The equation for calculating the number of clusters is shown in Equation (4).


$$\begin{array}{c} P_{T}=\left(\frac{\sum_{i\in l}P_{L_{i}}}{(P_{Li}\geq 1\overset{71}{\underset{L_{i}=0}{)}}}\right) \end{array}$$ (4)

**Fig 3:**
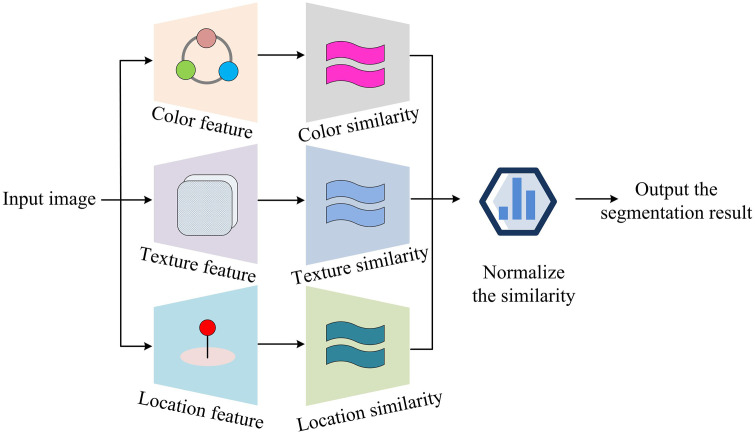
Multi-dimensional image segmentation method.

In Equation (4), $(P_{Li}\geq 1\overset{71}{\underset{L_{i}=0}{)}}$ represents the number of color feature values with a cluster number greater than or equal to 1, and $\sum_{i\in l}P_{L_{i}}$ represents the number of clusters in a single feature value. Multi-dimensional segmentation methods can integrate different feature types to complement and validate each other, thus improving segmentation accuracy and reasonability [19–20]. Therefore, the study uses color and texture as supplementary constraints to jointly determine the segmentation result through this multi-dimensional segmentation method (Fig 3).

As shown in Fig 3, the multi-dimensional image segmentation method first extracts the color, texture, and position distance features of the target image. Then, the similarity between the multi-dimensional features of different clusters is calculated. After normalizing the multi-feature similarity, the final similarity value is obtained and used as the basis for image segmentation. The color similarity between different clusters is calculated using Equation (5).


$$\begin{array}{c} d_{c}\left(p_{\text{1}},p_{\text{2}}\right)=\frac{\text{1}}{\text{2}}(S_{\text{1}}+S_{\text{2}})sin\left(\frac{\left|H_{\text{1}}-H_{\text{2}}\right|}{\text{2}}\right) \end{array}$$ (5)

In Equation (5), $p_{\text{1}},p_{\text{2}}$ represents the compared cluster group, and $H_{\text{1}}$, $S_{\text{1}}$, $H_{\text{2}}$, and $S_{\text{2}}$ represent the hue and saturation components of the compared clusters. $d_{c}$ represents the difference in the color feature dimension of the compared clusters. For distance similarity, the study uses Manhattan distance, with the formula shown in Equation (6).


$$\begin{array}{c} d_{ij}=\sum_{m=1}^{d}\left|x_{im}-x_{jm}\right| \end{array}$$ (6)

In Equation (6), m represents the vector dimension, and $d_{ij}$ represents the position distance between vectors $x\left(i_{\text{1}},i_{\text{2}},...,i_{m}\right)$ and $x\left(j_{\text{1}},j_{\text{2}},...,j_{m}\right)$. The texture histogram is directly constructed based on the grayscale values of the image, and the histogram features are formed by statistical analysis of the distribution of pixel grayscale values in each region. For texture feature similarity, this study calculates the intersection distance by adding the minimum values of texture histograms between different clusters. The smaller the value, the lower the texture similarity. The calculation of intersection distance is shown in Equation (7).


$$\begin{array}{c} d_{h}\left(H_{\text{1}},H_{\text{2}}\right)=\sum_{i=1}^{N}min\left(H_{\text{1}},H_{\text{2}}\right) \end{array}$$ (7)

In Equation (7), N represents the number of bins in the color concentration area. After obtaining the color, position, distance, and texture similarities of the clusters, the results are normalized to obtain a unified multi-dimensional similarity value. Finally, the multi-dimensional similarity values are summed to obtain the final similarity value. The normalization process is implemented using Equation (8).


$$\begin{array}{c} X_{norm}=\frac{X-X_{min}}{X_{max}-X_{min}} \end{array}$$ (8)

**Fig 4:**
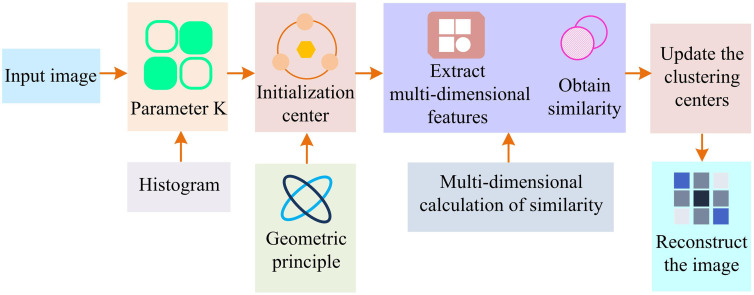
DM-K-means image compression method flow.

The Min-Max normalization method is a linear data standardization method that scales the original data proportionally to unify measurement standards [21–22]. In Equation (8), X, $X_{max}$, and $X_{min}$ represent the original similarity values, maximum similarity value, and minimum similarity value, respectively. Finally, the color, position distance, and texture similarities are added to form the final comprehensive similarity, which is used as the basis for K-means image segmentation. By integrating geometric principles, histograms, and multi-dimensional segmentation methods into K-means, the study creates a dynamic parameter acquisition and multi-dimensional segmentation image compression method called DM-K-means (Fig 4).

As shown in Fig 4, the proposed DM-K-means image compression method first determines the compression parameter, the number of clusters k, using the histogram-based dynamic parameter acquisition method. Then, the geometric principle method is used to compute the initial center. The color, texture, and position distance features of the image are extracted, and multi-dimensional similarity is calculated. Based on the multi-dimensional similarity, the image is clustered. Finally, the image is reconstructed based on the clustering results, completing the image compression task.

### 2.2. Construction of compression and optimization model for digital media pattern design

**Fig 5:**
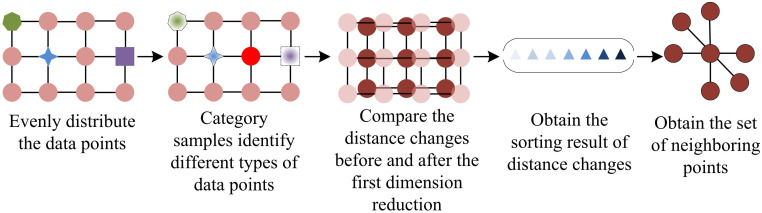
Nearest neighbor selection process.

The DM-K-means image compression method enables the compression of digital media patterns, allowing for better storage and transmission of patterns. However, image processing often encounters the “curse of dimensionality.” Excessive image data dimensions increase the computational complexity of the method, which impacts the overall model performance. Experts in various fields are exploring effective dimensionality reduction methods to facilitate efficient data sharing and utilization [23–24]. The LLE algorithm reduces dimensionality by reconstructing local linear relationships from high-dimensional space within a low-dimensional manifold. This method performs well in reducing computational complexity and has a wide range of applications. Therefore, the study applies the LLE algorithm for dimensionality reduction of image data. The LLE algorithm’s implementation steps are as follows: first, select m nearest neighbors for a given sample, then determine the weight coefficients for the relationship between the sample and its neighbors. Finally, based on these weight coefficients, the data points are reconstructed while preserving the local linear relationships between low-dimensional and high-dimensional spaces, achieving dimensionality reduction. However, the LLE algorithm is highly sensitive to the size of the neighborhood and the selection of nearest neighbors. Improper selection can affect the dimensionality reduction results. Therefore, the study improves the LLE algorithm’s neighborhood size and nearest neighbor selection to enhance the dimensionality reduction quality (Fig 5).

As shown in Fig 5, the improved nearest neighbor selection method adjusts effective distances to balance sparse and dense data points, ensuring a more uniform distribution. The method then introduces class samples to exclude data points from different classes, forming the first round of nearest neighbor data sets. Next, the original data points are compared with the first round of nearest neighbor data, and the changes in their position distances are sorted in ascending order. The data points with the smallest changes are selected as the second round of nearest neighbor data. Equation (9) describes the process of incorporating class information.


$$\begin{array}{c} {d^{\prime }}_{ij}=d_{ij}+\alpha max\left(d\right)\Lambda_{ij} \end{array}$$ (9)

In Equation (9), $d_{ij}$ is the initially changed distance, max(d) is the maximum distance between data points from different classes, $\Lambda_{ij}$ represents the data point’s class, α is an empirical parameter controlling data point spacing (set to 0.4 in this study), and ${d^{\prime }}_{ij}$ is the distance of data points integrated with class information. The distance is shown in Equation (10).


$$\begin{array}{c} d_{ij}\left(x_{i},x_{j}\right)=\frac{\|xi-x_{j}\|}{\sqrt{M\left(x_{i}\right)M\left(x_{j}\right)}} \end{array}$$ (10)

In Equation (10), $x_{i}$ and $x_{j}$ represent two measured data points, and $M\left(x_{i}\right)$ and $M\left(x_{j}\right)$ are the average distances between the two measured data points and other data points. The changes in distances between data points in high-dimensional and low-dimensional spaces are calculated according to Equation (11).


$$\begin{array}{c} \Delta_{ij}=\|d^{\prime }\left(x_{i},x_{ij}\right)-d^{\prime }\left(y_{i},y_{ij}\right)\| \end{array}$$ (11)

In Equation (11), $d^{\prime }\left(x_{i},x_{ij}\right)$ and $d^{\prime }\left(y_{i},y_{ij}\right)$ represent the distances between data points and neighboring points in high and low-dimensional spaces. $\Delta_{ij}$ represents the distance error between the two spaces. A smaller value of $\Delta_{ij}$ indicates that the topological structure differences between the dimensions are minimal. For neighborhood size selection, the study determines it using the popular curvature metric. In regions with high local curvature of data points, a larger neighborhood is chosen, and vice versa. The relationship between the curvature and the ratio of the position distance to the Euclidean distance is proportional. The process for measuring the local curvature of data points is shown in Equation (12).


$$\begin{array}{c} l\left(S_{i}\right)=\sum_{x_{ij},x_{ik}\in S_{t}}d_{g}\left(x_{ij},x_{ik}\right)/d_{c}\left(x_{ij},x_{ik}\right) \end{array}$$ (12)

In Equation (12), $d_{g}\left(x_{ij},x_{ik}\right)$ and $d_{c}\left(x_{ij},x_{ik}\right)$ are the position distance and Euclidean distance between any two points in the initial neighborhood $S_{i}$ of the data point. The indicator function and its average value in the high-dimensional space are calculated for all data points, with the expressions for the indicator function and its average value given in Equation (13).


$$\begin{array}{c} \left\{ \begin{array}{c} @l@\theta \left(S_{i}\right)=\frac{\text{1}}{l\left(S_{i}\right)}\\ \overset{―}{\theta }=\frac{\text{1}}{N}\sum_{\text{1}}^{N}\theta \left(S_{i}\right) \end{array}\right. \end{array}$$ (13)

In Equation (13), i=1,2,3,...,N. The final neighborhood size of the data points is determined dynamically, as shown in Equation (14).


$$\begin{array}{c} K^{\prime }=\frac{\theta \left(S_{i}\right)}{\overset{―}{\theta }}K \end{array}$$ (14)

**Fig 6:**
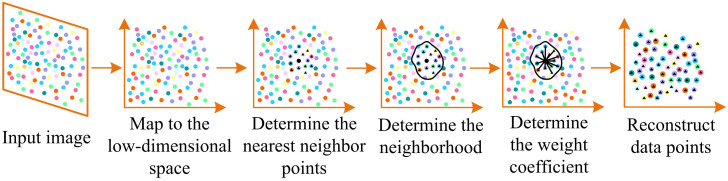
DAF-LLE algorithm flowchart.

In Equation (14), K is the initially preset neighborhood size, and $K^{\prime }$ is the final computed neighborhood size. The study integrates the improved nearest neighbor and neighborhood size selection methods into the original LLE algorithm, designing a dynamic neighborhood selection LLE algorithm, named DAF-LLE (Fig 6).

As shown in Fig 6, the DAF-LLE algorithm first maps high-dimensional image data to a low-dimensional space and dynamically optimizes the neighborhood structure for dimensionality reduction. In the nearest neighbor selection stage, the algorithm adjusts the spacing between data points based on category information, prioritizes selecting samples of the same class as candidate neighbors, and then determines the final set of neighbors based on the principle of minimizing distance changes. In the adaptive stage of neighborhood size, the algorithm dynamically adjusts the neighborhood range of each data point based on local curvature evaluation, assigning larger neighborhoods to areas with high curvature. Finally, in the weight calculation and reconstruction stage, the weight matrix is calculated based on the selected neighbors, and the optimal weights are obtained by minimizing the reconstruction error, and data dimensionality reduction is completed in a low-dimensional space. The loss function is shown in Equation (15).


$$\begin{array}{c} \varepsilon \left(w\right)={\sum_{i=1}^{N}||x_{i}-\sum_{j=1}^{k}w_{ij}x_{ij}||}^{\text{2}} \end{array}$$ (15)

**Fig 7:**
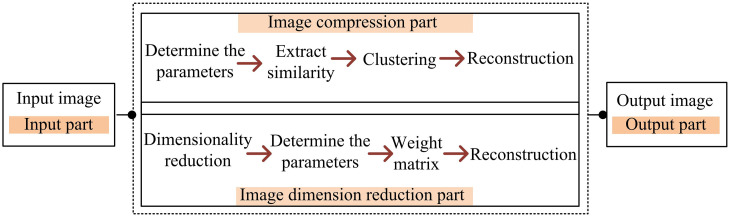
K-means-LLE model framework.

In Equation (15), N represents the number of data points, and $W_{ij}$ denotes the contribution of the j -th data point to reconstructing the i -th data point. If $x_{j}$ is not a neighboring point of $x_{i}$, then $w_{ij}=0$. Finally, the study combines the DM-K-means image compression method and the DAF-LLE dimensionality reduction algorithm to build a digital media pattern compression model based on K-means and LLE, named K-means-LLE (Fig 7).

Fig 7 displays the processing framework of the K-means-LLE model for patterns. In the compression process, the DM-K-means stage achieves initial compression by reducing the number of colors, but its output dimension is still relatively high, especially when processing large-sized images, which can bring significant storage and computational overhead. Therefore, after DM-K-means compression, the DAF-LLE dimensionality reduction step is introduced to map the high-dimensional feature space output by DM-K-means to a carefully constructed low dimensional manifold space. The high-dimensional features here mainly refer to the feature set composed of color, texture, and spatial position information. DAF-LLE preserves the local neighborhood structure of sample points from the high-dimensional feature space while removing redundant information and noise in the low-dimensional embeddings, further reducing the data required to represent compressed images while maximizing visual fidelity. This dimensionality reduction operation improves the overall compression pipeline, not only reducing the bit rate required for subsequent storage and transmission of these compressed features, but also accelerating any possible subsequent processing tasks, thereby achieving a better balance between compression ratio and computational efficiency. The pseudocode of the proposed K-means LLE algorithm is shown in Table 1.

**Table 1:** Pseudo code of K-means LLE algorithm.

Algorithm: K-means-LLE (Image Compression and Dimensionality Reduction)
**Input:**
input_image // Raw input image
**Output:**
compressed_image// Final compressed and dimensionality-reduced image representation
**BEGIN:**
// **Step 1: Determine optimal cluster number k based on HSI color histogram** k ← detect_peaks(convert_to_HSI)
**// Step 2: Initialize cluster centers using geometric principles** centers ← initialize_centers(input_image, k)
**// Step 3: Extract multi-dimensional features (color, texture, spatial position)** color_features ← extract_color_features(input_image) texture_features ← extract_LBP_texture(input_image) position_features ← extract_pixel_positions(input_image)
**// Step 4: Compute multi-dimensional similarity matrix** color_sim ← compute_color_similarity(color_features) texture_sim ← compute_texture_similarity(texture_features) position_sim ← compute_position_similarity(position_features) similarity_matrix ← normalize_and_fuse(color_sim, texture_sim, position_sim)
**// Step 5: Perform improved K-means clustering for preliminary compression** quantized_image ← DM_K_means(input_image, k, centers, similarity_matrix)
**// Step 6: Apply improved LLE for further dimensionality reduction** compressed_representation ← DAF_LLE(quantized_image) **END IF**
**END FOR**
// RETURN compressed image representation
RETURN compressed_representation
**END**

### 2.3. Experimental design

In the DM-K-means method, the number of clusters k is dynamically determined through color histogram analysis, with a threshold set at 15% of the total number of feature values. In the DAF-LLE algorithm, the empirical parameter α is determined to be 0.4 after grid search optimization. The neighborhood size is dynamically adjusted based on local curvature, with an initial preset neighborhood size of 12, and adaptively calculated using formula (14). The number of nearest neighbors during the construction of the weight matrix is set to 8. All parameters were determined through cross-validation in preliminary experiments to ensure that the model maintains stable performance on different types of images. To validate the performance of the K-means-LLE model proposed in this study, performance verification was carried out through both ablation and comparative experiments. The ablation experiment primarily verifies the effectiveness of the model’s internal improvements, comparing K-means, LLE, and their combined versions by controlling variables to confirm the contribution of each component to the final performance. The comparative experiment evaluates the comprehensive performance of the model in a wider competitive environment, comparing the complete K-means-LLE model against industry-standard methods such as Joint Photographic Experts Group (JPEG), High Efficiency Video Coding (HEVC), and Efficient Learning Image Compression (ELIC) to demonstrate its overall advantages. These two experiments jointly validated the value of the model from different dimensions – the ablation experiment ensured its design rationality, and the comparative experiment proved its actual competitiveness. For the ablation experiments, the ADE20K image dataset was used, which is an open scene understanding dataset divided into a training set and a validation set. The training set contains 25,574 patterns, and the validation set contains over 2,000 patterns. The dataset covers patterns from over 300 different scene categories, including sports, daily life, and work. For the comparative experiments, the ImageNet dataset was used, which is currently the world’s largest database. It is a collection of patterns based on the WordNet hierarchy and contains 14 million high-resolution patterns across more than 20,000 categories, formatted in PASCAL VOC. The ablation experiments compared the image compression performance of the K-means, LLE, and K-means-LLE models. The comparative experiments evaluated the performance of the K-means-LLE model by comparing it with three other models: Joint Photographic Experts Group (JPEG), High Efficiency Video Coding (HEVC), and Efficient Learned Image Compression (ELIC). Both the ablation and comparative experiments were conducted in the same experimental environment (Table 2).

**Table 2:** Experimental environment parameters.

Disposition	Argument
CPU	AMD Ryzen 5
GPU	NVIDIA RTX 3060
Memory	32GB
Operating system	Windows 11
Programming language	Python 4.0
IDE	VS Code

After the ablation experiments validated the effectiveness of the proposed optimization, the study proceeded with comparative experiments on the practical application of the K-means-LLE model. The experiments were conducted in the same environment as described in Table 2, using the ImageNet dataset. The first step involved a comprehensive evaluation of the four models. “Comprehensive” here refers to whether the models could achieve both high compression ratios and low image differences after compression.

## 3. Results

### 3.1. Experimental setup and ablation study

To evaluate the sensitivity of the model to key parameters, this study conducted testing on three parameters: cluster number k, empirical parameter α, and neighborhood size. The experiment was conducted on 100 images in the ADE20K dataset, with only one parameter adjusted at a time and the remaining parameters fixed as default values. The changes in compression ratio and Peak Signal-to-Noise Ratio (PSNR) were recorded (Table 3).

**Table 3:** Sensitivity analysis results of three parameters.

Parameter	Value	Compression ratio/%	PSNR/dB
k	20	88.5	38.6
	100	76.7	44.9
	150	65.5	45.1
α	0.3	85.8	46.3
	0.5	85.4	46.6
	0.7	83.1	44.2
Neighborhood size	5	84.3	45.1
	12	86.2	46.7
	20	85.1	45.9

**Fig 8:**
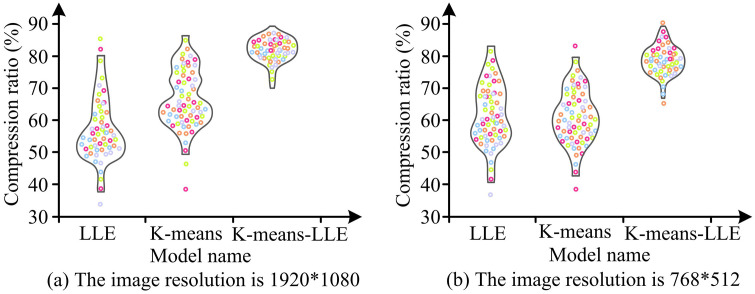
Comparison results of compression ratios of three models at different image resolutions.

Table 3 indicates that the k value significantly impacts performance. When the k value increases from 20 to 150, the PSNR increases from 38.6 dB to 45.1 dB, while the compression ratio decreases from 88.5% to 65.5%. The parameter α yields stable performance within the range of 0.3–0.5, but it decreases slightly beyond this range. The influence of neighborhood size on model performance is relatively small. Based on the experimental environment in Table 2 and the ADE20K image dataset, the study first conducted a comparison of the compression ratios of the K-means, LLE, and K-means-LLE models. The experiment involved compressing 100 patterns of two different resolutions using three image compression models (Fig 8).

**Fig 9:**
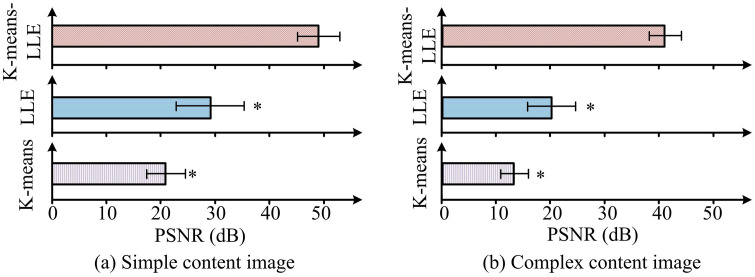
Comparison results of PSNR between three models on simple content images and complex content images. Note: “*” indicates a significant difference compared to K-means-LLE, p < 0.05.

From Fig 8, it can be seen that when the resolution of the compressed image is 1920 × 1080, the compression ratio for LLE ranged from 48% to 53%, with a maximum of 79% and a minimum of 38%, with a few outliers. For K-means, the compression ratio ranged from 57% to 62%, with a maximum of 83% and a minimum of 48%. The proposed K-means-LLE model had a compression ratio ranging from 77% to 84%, with no outliers. For high-resolution patterns, K-means-LLE performed significantly better than the basic models. When the compressed patterns were of low resolution (768 × 512), K-means-LLE still demonstrated superior compression performance, with a median compression ratio of 79%, outperforming LLE (61%) and K-means (62%). These results indicate that the compression ratio of K-means-LLE is less affected by image resolution, and regardless of the image quality, K-means-LLE can still perform high-quality compression. Moreover, these results demonstrate that the proposed optimized model provides a significant improvement in compression performance over the basic models. After the compression ratio experiments, the study continued by comparing the PSNR of LLE, K-means, and K-means-LLE. PSNR quantifies the compression effect of the models by comparing the pixel value differences between the original and compressed patterns. A higher PSNR indicates that the difference between the compressed and original patterns is smaller, meaning better compression quality. The experiments selected two groups of patterns with different content complexities: one group containing patterns with detailed textures like leaves and hair, and another group containing simple patterns with a single color, like the sky and blackboards. All experiments were independently repeated 10 times, and the results were reported in the form of mean ± standard deviation. Paired *t*-test was used for statistical significance analysis, with a significance level set at *p* < 0.05 (Fig 9).

**Fig 10:**
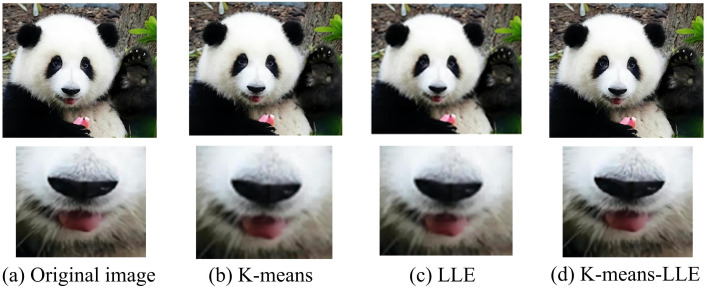
Visual quality comparison between the original image and the output results of three compression methods (Source: http://mbd.baidu.com/newspage/data/dtlandingsuper?nid=dt_5615633959172790718).

From Fig 9(a), it can be seen that when the compressed image is of simple content, the average PSNR of K-means-LLE was 48dB, meaning that the difference between the original and compressed patterns was almost imperceptible to the human eye. The average PSNR for LLE was 28dB, and for K-means, it was 21dB, indicating that the difference between the compressed and original patterns was noticeable. In contrast, K-means-LLE had the least perceptible difference when processing simple content patterns. From Fig 9(b), it can be seen that when compressing complex content patterns, the average PSNR of all three models decreased to some extent. K-means-LLE maintained an average PSNR of 41 dB, with minimal distortion. LLE had an average PSNR of 20dB, indicating considerable distortion during compression. K-means had an average PSNR of 13dB, suggesting more significant distortion, with visible quality degradation. These results show that, regardless of the complexity of the image content, K-means-LLE can perform high-quality compression. In the final part of the ablation experiments, the study visually compared the compression effects of the three models (Fig 10).

As seen in Fig 10(b), after compressing the original image using K-means, it was difficult for the human eye to detect any differences. However, upon zooming in, some blurring was visible, and the image quality showed a slight decline. In Fig 10(c), when the image was compressed using LLE, noticeable differences in clarity were observed, especially in the detailed areas where blurring was more severe. This indicates that LLE caused some loss of information during the compression process. In Fig 10(d), when K-means-LLE was used, no significant difference in image quality was observed, and the details were preserved with adequate clarity. In summary, K-means-LLE outperforms the basic models, K-means and LLE, significantly in terms of image compression performance, demonstrating that the proposed optimization is effective.

### 3.2. Verification of model’s practical application performance

**Fig 11:**
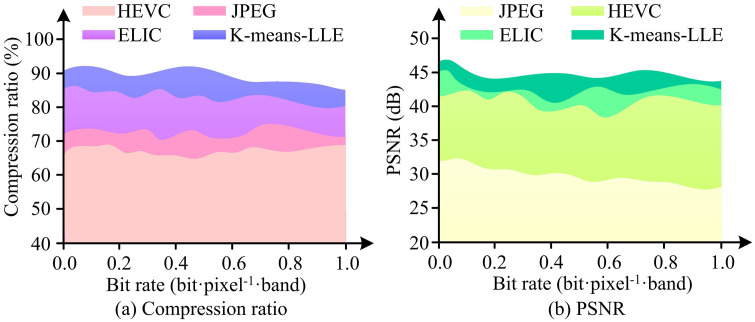
Comparison results of the compression ratio and PSNR index of four compression models.

The comprehensive experiments used both the compression ratio and PSNR to evaluate the models’ performance (Fig 11).

**Fig 12:**
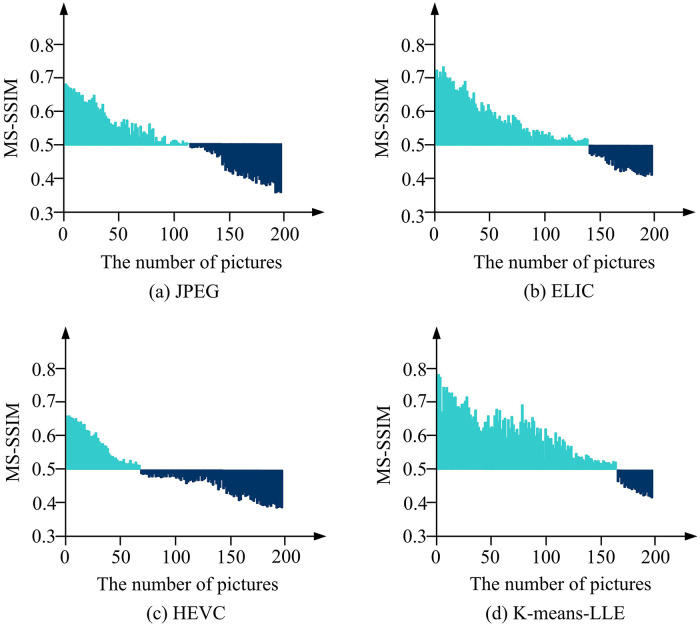
Comparison results of four models on MS-SSIM metrics.

From Fig 11 (a) and 11(b), it can be seen that as the bit rate increased, JPEG’s maximum compression ratio was 75.2%, with a PSNR of 33.4dB. HEVC had a maximum compression ratio of 69.7% and a PSNR of 43.1dB. While HEVC had a slightly lower compression ratio than JPEG, its PSNR value was higher, suggesting that HEVC outperforms JPEG in terms of image comprehensiveness, maintaining better image quality with only a slight sacrifice in compression rate. ELIC had higher values for both compression ratio and PSNR than both JPEG and HEVC, with a maximum compression ratio of 86.1% and a PSNR of 45.3dB, indicating superior comprehensiveness. In comparison, the K-means-LLE model achieved the best performance in both compression ratio and PSNR, with a maximum compression ratio of 91.1% and a PSNR of 47.2dB. This demonstrates that K-means-LLE has both a high compression ratio and a high PSNR, providing an excellent balance between compression effect and image quality. Next, the study used the Multi-Scale Structural Similarity Index (MS-SSIM) to compare the four models. MS-SSIM evaluates the structural similarity of the patterns after compression by considering the luminance, contrast, and structure. A higher MS-SSIM indicates that the compressed image is more similar to the original image, and thus the distortion is smaller (Fig 12).

**Fig 13:**
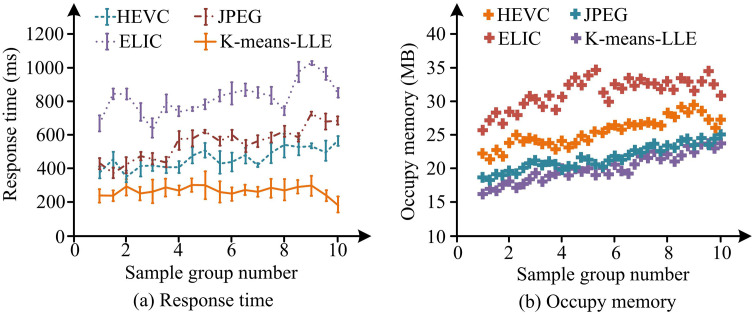
Comparison of response time and memory usage when four models process 100 images simultaneously.

From Fig 12(a), JPEG’s MS-SSIM ranged from a maximum of 0.69 to a minimum of 0.36. As the number of patterns increased to 116, the MS-SSIM dropped below 0.5. In Fig 12(b), ELIC had a maximum MS-SSIM of 0.72 and a minimum of 0.39, dropping below 0.5 when the number of compressed patterns reached 148. Fig 12(c) shows that HEVC’s MS-SSIM was mostly below 0.5, with a maximum of 0.67 and a minimum of 0.37. In contrast, Fig 12(d) shows that K-means-LLE had the highest MS-SSIM values, with a maximum of 0.79 and a minimum of 0.41. This data demonstrates that K-means-LLE preserves more of the structural integrity of the original patterns during compression, providing superior compression performance. After verifying the compression performance of K-means-LLE, the study continued by testing its practical applicability. The first step involved comparing the response time and memory usage of the four models. The experiment used 10 groups of patterns, each containing 100 patterns from different categories. The study verified the response time and memory usage when all four models processed 100 patterns simultaneously (Fig 13).

**Fig 14:**
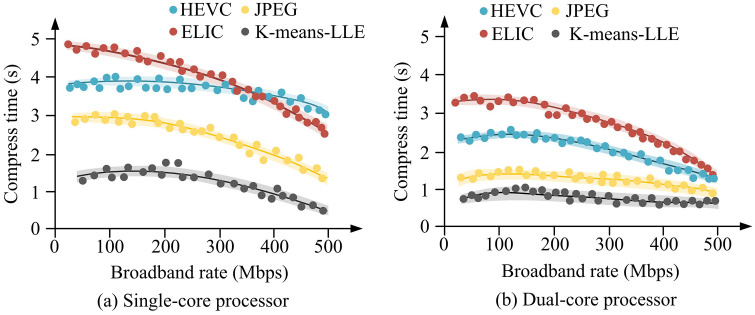
Comparison of the processing time of four models on single-core processors and dual-core processors with different bandwidths.

As shown in Fig 13(a), for the 10 groups of experimental subjects, the response time of ELIC was the shortest at 612ms and the longest at 1012ms, indicating relatively long response times. For the HEVC compression model, the shortest and longest response times were 322ms and 571ms, respectively, showing relatively short response times. The shortest and longest response times for JPEG were 384ms and 732ms, respectively, placing its response time between that of HEVC and ELIC. The response times for K-means-LLE were the shortest at 189ms and the longest at 294ms, clearly faster than the other three models. The small difference between the shortest and longest response times suggests that K-means-LLE not only processed multiple patterns quickly but also had stable response times. Fig 13(b) presents the memory usage results, showing that K-means-LLE occupied the least memory among the four models, significantly less than HEVC and ELIC, and slightly less than JPEG. When compressing 100 patterns simultaneously, the minimum and maximum memory usage for ELIC, HEVC, and JPEG were 25.1MB, 20.8MB, and 18.2MB, and 34.8MB, 27.9MB, and 25.0MB, respectively. For K-means-LLE, the minimum and maximum memory usage were 16.1MB and 23.2MB. These results indicate that K-means-LLE outperformed the comparison models in both response time and memory usage when compressing a large number of patterns simultaneously, making it efficient and practical for handling multiple processing tasks. Next, the study verified the dependence of the four models on the operating environment through comparative experiments. The experiments were conducted in two steps. The first step tested the duration required to process 100 patterns with increasing bandwidth using a single-core processor. The second step validated the same process using a dual-core processor (Fig 14).

Fig 14 reflects the varying processing times for the four models on single-core and dual-core processors and also highlights their dependence on computing resources. As shown in Fig 14(a), with a single-core processor, when the bandwidth was 100Mbps and 500Mbps, the processing time for ELIC decreased from 4.95s to 2.84s. For HEVC, the processing times were 3.75s and 3.21s, and for JPEG, the longest and shortest processing times were 2.98s and 1.59s, respectively. For K-means-LLE, the processing time was 1.22s at 100Mbps and 0.34s at 500Mbps. Fig 14(b) shows the results for the dual-core processor. In this case, the processing times for all four models were reduced. With a bandwidth of 100Mbps, the processing times for ELIC, HEVC, JPEG, and K-means-LLE were 3.25s, 2.42s, 1.59s, and 0.97s, respectively. The processing times were reduced by 0.7s, 0.79s, 1.39s, and 0.25s compared to the single-core processor. Notably, K-means-LLE had the shortest processing time on both the single-core and dual-core processors, with the smallest difference in processing time between the two. These experimental results demonstrate that K-means-LLE has a lower dependence on computing resources and can efficiently run on a variety of machines, indicating its broad applicability.

## 4. Discussion

To meet the high demands of modern digital media patterns for image compression technology, this study proposed an image compression model, K-means-LLE, based on K-means clustering and dimensionality reduction techniques. Ablation experiments and comparative tests verified the K-means-LLE model’s performance. The results of the ablation experiment showed that K-means-LLE achieved a compression ratio of 84% and a PSNR of 48 dB, indicating that it can maintain high visual quality while significantly reducing data volume. This performance improvement is mainly due to the dynamic clustering parameter selection and multi-dimensional feature fusion strategy, effectively overcoming the limitations of traditional methods in initial center selection and neighborhood sensitivity issues [25]. Unlike Rui et al.’s improved approach of using hierarchical clustering to enhance computational efficiency [26], the dynamic parameter and multi-dimensional feature fusion mechanism proposed in this study focuses more on ensuring and enhancing compression quality through parameter adaptation and feature complementarity while improving efficiency. The dimensionality reduction optimization method used by Oh et al. [27] focuses on reducing computational resource overhead. In contrast, the DAF-LLE algorithm proposed in this study focuses more on maintaining the local manifold structure of the data to ensure visual fidelity after dimensionality reduction, thus achieving a better balance between resource consumption and reconstruction quality. In practical applications, K-means-LLE achieved a compression ratio of 91.1% and a PSNR of 47.2 dB, demonstrating its ability to maintain high-quality compression performance even when processing complex images. The slight difference in PSNR values between ablation experiments and practical application tests (48 dB and 47.2 dB) is mainly due to the different datasets used in the two experiments. 48 dB is the test result on the ADE20K image dataset, while 47.2 dB is the test result on the ImageNet dataset. This result is of great significance in practical applications, as a high compression ratio means that about 90% of storage space and transmission bandwidth can be saved, especially for network transmission or resource-limited mobile devices. A high PSNR value ensures that the compressed image has almost no visual distortion, making it particularly suitable for scenes that require high detail preservation, such as art and design, high-precision printing, and other fields [28]. Although Kim’s rate distortion-based method also aims to balance compression ratio and quality [29], the K-means LLE model proposed in this study achieves better overall performance in key indicators such as PSNR and compression ratio through a collaborative architecture of clustering and dimensionality reduction. In the multi-scale structural similarity experiments, K-means-LLE achieved the highest structural similarity of 0.79, attributed to the use of multi-dimensional feature similarity calculations. Similar to Lin’s approach of integrating multi-scale features to enhance the robustness of drone tracking [30], this study integrates color, texture, and positional features in multi-dimensional image segmentation. When handling patterns of varying complexities, K-means-LLE exhibited the shortest response time of 189 ms, with memory usage as low as 16.1 MB, demonstrating its efficiency and stability in handling a large number of image tasks. This feature makes the K-means LLE model particularly suitable for real-time or near-real-time application scenarios, such as online image editing platforms, multimedia messaging services, etc. In addition, the model exhibits shorter execution time and smaller performance fluctuations on both single-core and dual-core processors, indicating its low dependence on hardware resources and good cross-platform applicability [31]. Furthermore, K-means-LLE displayed fast processing speeds across different processor specifications, which can be attributed to the reasonable design of the K-means-LLE model. Regarding the rate issue in image compression, Zhang saved decoding time by decoupling the structure, thereby shortening the image compression duration [32]. Kim, on the other hand, reduced memory usage by dynamically scheduling the workload of the model [33]. The experimental results demonstrated that the K-means-LLE model provides superior image compression performance and practicality, offering more professional technical support for digital media workers and contributing to the intelligent and diversified development of digital media. The K-means LLE model in this study demonstrates comparable or even better efficiency with its lightweight architecture that combines clustering and dimensionality reduction.

In summary, the K-means LLE model not only achieves a good balance between compression efficiency and quality but also demonstrates significant storage and bandwidth saving potential in practical applications, adapting to various complex scenarios. The successful practice of this model provides strong support for the intelligent processing and efficient transmission of digital media content, which helps to promote the development of related industries towards a more efficient and environmentally friendly direction. However, this study did not conduct in-depth transferability validation for specific domain images, such as medical imaging, satellite images, etc., resulting in unclear applicability and performance of the model in fields with unique imaging characteristics and professional requirements. Therefore, in future work, systematic transferability validation should be conducted on more specialized datasets, such as the NIH ChestX-ray14 in the field of medical imaging and the EuroSAT dataset in the field of remote sensing images. At the same time, targeted domain adaptation techniques are introduced to optimize the feature extraction module, in order to enhance the model’s generalization ability in professional domains.

## 5. Conclusion

Digital media patterns, due to their rich content and vibrant color representation, have gained attention in various fields such as virtual reality, film, and gaming. However, substantial redundant information limits the storage and transmission of digital media patterns, creating an urgent need for compression. Traditional image compression methods struggle to balance compression efficiency and quality. To address this, this study combines K-means clustering and LLE dimensionality reduction techniques to propose a new digital media pattern compression model. This model dynamically determines the number of clusters and initial centers, utilizes multi-dimensional features for accurate segmentation, and applies improved dimensionality reduction algorithms to process high-dimensional features. Ultimately, while maintaining high-quality visual performance, it achieves higher compression ratios, faster processing speeds, and lower memory usage. In the ablation experiments, the compression ratio and image quality before and after compression were superior to those of the baseline models, demonstrating the effectiveness of the optimizations and improvements made to the model. In practical application experiments, the results showed that the model was able to maintain the quality of the compressed patterns while ensuring a high compression ratio, with minimal structural differences between the patterns before and after compression. When handling a large number of patterns, the model exhibited faster response times and lower memory usage than the comparison models, while also demonstrating a favorable dependence on the processor. Overall, the proposed digital media pattern compression model exhibited excellent compression performance and practicality, meeting the high standards required by the digital media industry for image compression.

## Supporting information

S1 File Minimal Data Set Definition. (DOC)
