# Supplementary material for: Digital media pattern design compression and optimization method based on K-means clustering and LLE dimensionality reduction
Source: PLoS One. 2026 Jun 16;21(6):e0350623. doi: 10.1371/journal.pone.0350623 (PMC13271481; doi:10.1371/journal.pone.0350623)
Supplement: S1 File — (DOC) [file pone.0350623.s001.doc]

**Figure 8. Comparison results of compression ratios of three models at different image resolutions**

(a) The image resolution is 1920*1080

| Compression ratios | LLE | K-means | K-means-LLE |
| --- | --- | --- | --- |
| Maximum/% | 79 | 83 | 88 |
| Minimum/% | 38 | 48 | 71 |

(b) The image resolution is 768*512

| Compression ratios | LLE | K-means | K-means-LLE |
| --- | --- | --- | --- |
| Maximum/% | 82 | 80 | 89 |
| Minimum/% | 40 | 44 | 68 |

**Figure 9. Comparison results of PSNR between three models on simple content images and complex content images**

| Image | LLE | K-means | K-means-LLE |
| --- | --- | --- | --- |
| Simple content image | 28 dB | 21 dB | 48 dB |
| Complex content image | 20 dB | 13 dB | 41 dB |

**Figure 11. Comparison results of compression ratio and PSNR index of four compression models**

(a) Compression ratio (%)

| Method | Bit rate (bit·pixel^-1^·band) | | | | |
| --- | --- | --- | --- | --- | --- |
|  | 0.2 | 0.4 | 0.6 | 0.8 | 1.0 |
| HEVC | 67.1 | 65.8 | 66.7 | 66.9 | 69.7 |
| JPEG | 72.5 | 71.6 | 70.8 | 74.6 | 71.5 |
| ELIC | 84.2 | 83.6 | 82.4 | 82.3 | 80.3 |
| K-means-LLE | 89.9 | 92.1 | 89.0 | 87.8 | 85.6 |

(b) PSNR (dB)

| Method | Bit rate (bit·pixel^-1^·band) | | | | |
| --- | --- | --- | --- | --- | --- |
|  | 0.2 | 0.4 | 0.6 | 0.8 | 1.0 |
| HEVC | 41.7 | 39.2 | 37.9 | 41.8 | 40.0 |
| JPEG | 31.1 | 30.0 | 28.8 | 28.5 | 27.8 |
| ELIC | 42.0 | 40.7 | 42.8 | 43.0 | 43.1 |
| K-means-LLE | 44.2 | 45.0 | 44.6 | 45.0 | 43.9 |

Figure 12. Comparison results of four models on MS-SSIM metrics

| Method | The number of pictures | | | |
| --- | --- | --- | --- | --- |
|  | 50 | 100 | 150 | 200 |
| HEVC | 0.53 | 0.47 | 0.43 | 0.37 |
| JPEG | 0.56 | 0.51 | 0.42 | 0.36 |
| ELIC | 0.62 | 0.54 | 0.47 | 0.39 |
| K-means-LLE | 0.66 | 0.62 | 0.53 | 0.41 |

**Figure 13. Comparison of response time and memory usage when four models process 100 images simultaneously**

(a) Response time (ms)

| Method | Sample group number | | | | |
| --- | --- | --- | --- | --- | --- |
|  | 2 | 4 | 6 | 8 | 10 |
| HEVC | 362 | 409 | 441 | 542 | 571 |
| JPEG | 422 | 587 | 598 | 625 | 691 |
| ELIC | 845 | 752 | 854 | 758 | 841 |
| K-means-LLE | 294 | 282 | 249 | 281 | 189 |

(b) Occupy memory (MB)

| Method | Sample group number | | | | |
| --- | --- | --- | --- | --- | --- |
|  | 2 | 4 | 6 | 8 | 10 |
| HEVC | 24.1 | 23.4 | 26.2 | 28.1 | 27.4 |
| JPEG | 19.6 | 20.1 | 22.0 | 24.4 | 25.0 |
| ELIC | 24.1 | 30.7 | 32.6 | 32.8 | 31.1 |
| K-means-LLE | 17.8 | 19.4 | 20.2 | 22.1 | 23.2 |

**Figure 14. Comparison of processing time of four models on single core processors and dual core processors with different bandwidths**

**(a) Single-core processor**

| Method | Broadband rate (Mbps) | | | | |
| --- | --- | --- | --- | --- | --- |
|  | 100 | 200 | 300 | 400 | 500 |
| HEVC | 3.75 | 3.74 | 3.69 | 3.46 | 3.21 |
| JPEG | 2.98 | 2.84 | 2.43 | 2.00 | 1.59 |
| ELIC | 4.95 | 4.38 | 3.96 | 3.41 | 2.84 |
| K-means-LLE | 1.22 | 1.21 | 1.18 | 0.98 | 0.34 |

(b) Dual-core processor

| Method | Broadband rate (Mbps) | | | | |
| --- | --- | --- | --- | --- | --- |
|  | 100 | 200 | 300 | 400 | 500 |
| HEVC | 2.42 | 2.37 | 2.09 | 1.78 | 1.36 |
| JPEG | 1.59 | 1.41 | 1.30 | 1.19 | 0.98 |
| ELIC | 3.25 | 3.17 | 2.83 | 2.32 | 1.39 |
| K-means-LLE | 0.92 | 0.81 | 0.73 | 0.70 | 0.69 |
